# Rate and factors associated with surgical site infection following aseptic revision fixation of orthopaedic trauma injuries

**DOI:** 10.1007/s00590-023-03573-3

**Published:** 2023-05-18

**Authors:** N. R. Heinz, N. D. Clement, R. N. Young, A. D. Duckworth, T. O. White, S. G. Molyneux

**Affiliations:** 1 Royal Infirmary of Edinburgh, Little France, Edinburgh, EH16 4SA UK; 2 University of Edinburgh, Edinburgh, UK

**Keywords:** Infection, Revision, SSI, Trauma

## Abstract

**Purpose:**

The primary aim of this study was to define the rate of infection following revision of fixation for aseptic failure. The secondary aims were to identify factors associated with an infection following revision and patient morbidity following deep infection.

**Methods:**

A retrospective study was undertaken to identify patients who underwent aseptic revision surgery during a 3-year period (2017–2019). Regression analysis was used to identify independent factors associated with SSI.

**Results:**

Eighty-six patients were identified that met the inclusion criteria, with a mean age of 53 (range 14–95) years and 48 (55.8%) were female. There were 15 (17%) patients with an SSI post revision surgery (*n* = 15/86). Ten percent (*n* = 9) of all revisions acquired a ‘deep infection’, which carried a high morbidity with a total of 23 operations, including initial revision, being undertaken for these patients as salvage procedures and three progressed to an amputation. Alcohol excess (odds ratio (OR) 1.61, 95% CI 1.01–6.36, *p* = 0.046) and chronic obstructive pulmonary disease (OR 11.1, 95% CI 1.00–133.3, *p* = 0.050) were independently associated with an increased risk of SSI.

**Conclusion:**

Aseptic revision surgery had a high rate of SSI (17%) and deep infection (10%). All deep infections occurred in the lower limb with the majority of these seen in ankle fractures. Alcohol excess and COPD were independent risk factors associated with an SSI and patients with a history of these should be counselled accordingly.

**Level of Evidence:**

Retrospective Case Series, Level IV.

## Introduction

Revision for aseptic failure of fixation of orthopaedic trauma injuries and the risk of associated complications is not well understood. Following aseptic revision of an arthroplasty the infection rate is well established as being over twice that associated with the primary surgery [1]. However, there is a paucity of literature identifying the infection rates following aseptic revision trauma operations for both upper and lower limb trauma. Risk factors for surgical site infection (SSI) following primary trauma procedures have been previously described as smoking, open fractures, diabetes, obesity, alcohol usage, surgical time, body mass index (BMI), gender, length of hospital stay and delay to primary surgery [2–4].

There are numerous reports in the literature that demonstrate re-operation rates on distal radius fractures, for example, are between 3.8% and 10% [5, 6]. Similarly the range of reported re-operation rates for ankle fracture fixation is between 8.7% and 12% [5, 7]. These rates are relatively high and include both septic and aseptic revision. A small number of studies have assessed infection rates post revision surgery as a subsection within their results, however, these are for specific procedures only resulting in small numbers and it is therefore difficult to extrapolate these results to other injuries. For example, patients undergoing revision procedures for tibial plateau mal-union were noted to have a 12% infection rate after aseptic revision surgery, however it could be argued this is not a failure of fixation [8]. Another study quoted a 3% infection rate following revision of femoral exchange nailing for aseptic non-union [9].

More data are required on the risks associated with revision trauma fixation surgery in order to inform surgeons and patients regarding the risks and benefits of an aseptic revision procedure.

The primary aim of this study was to define the rate of infection following revision fixation for aseptic failure. The secondary aims were to identify factors associated with infection following revision trauma fixation for aseptic failure as well as patient morbidity following deep infection.

## Methods

A retrospective study was undertaken. Formal ethical approval was not required as no patient contact was undertaken. No formal funding was received. The study centre is the only acute trauma service available to a population of over 800 000 people. A database was retrospectively compiled from the local Operating Schedule System (ORSOS) for patients over a three-year period between January 2017 and December 2019 at the study centre, which allowed for a minimum following up of 2-years. All patients with two or more operative records were screened. Inclusion criteria were any patient over 13 years of age undergoing two or more operations for the same injury. Exclusion criteria were an open fracture at the time of the first injury, patient under 13 years of age, patients out of the catchment population, patients with either superficial or deep infection after the primary procedure and those that were found to be infected at the first revision procedure via tissue cultures. The remainder of the patients had no infection at the time of the first revision procedure. Revision included patients who had further surgery due to complications, such as pain, mal-union, aseptic non-union or mechanical failure. Patients revised due to pain were those with prominent or uncomfortable underlying metalwork, and therefore metalwork removal was included in this cohort.

Data were retrospectively collected electronically using the study centre’s electronic TrakCare system (InterSystems Corp, Cambridge, Massachusetts). These included patient demographics, co-morbidities, initial fracture type, operative procedures undertaken, time between all surgical procedures and reasons for revision surgery. Patients with any documentation of smoking around the time of injury were deemed to be smokers. Patients with an alcohol intake documented of over 14 units per week as per United Kingdom Government guidelines [10] or those with a diagnosis of ‘alcohol excess’ satisfied the criteria for alcohol as a risk factor. Fractures were defined using the Arbeitsgemeinschaft für Osteosynthesefragen (AO) classification. Scottish Index of Multiple Deprivation (SIMD) was defined as the relative measure of deprivation [11] and split into quintiles with ‘1’ being the least most deprived and ‘5’ being the least deprived. Radiographs were reviewed using Carestream Picture Archiving and Communication System (PACS) system (Carestream Health, Rochester, New York).

### Outcomes

Our primary outcome was infection rate after revision surgery and our secondary outcomes were risk factors for infection after revision trauma surgery. ‘Deep infection’ was defined by positive microbiology taken during surgery. ‘Superficial infection’ was defined as clinical cellulitis surrounding the surgical wound with no proven ‘deep infection’. We used the definition as stated by the Centre for Disease Control for surgical site infection which is an ‘ infection of the incision or organ or space that occur after surgery’ [12].

### Follow-up

All patients were followed up at standard intervals of 2 and 6 weeks. Follow-up thereafter was as per treating surgeons’ discretion based on complications encountered. Consultation details were collected via the study centre’s electronic TrakCare system (a healthcare information system) retrospectively. A short case series of patients who had acquired a deep infection was conducted in order to highlight the gravity of complications encountered and is shown in Table 1.

**Table 1:** Case series summary table of patients who acquired deep infections and their final outcomes

No	Fracture/injury	Age/gender	BMI	Co-morbidities	Laboratory results (infected presentation)	Outcome	Total operations (after primary fixation)
5	Ankle	54F	22	COPD, depression	WCC 12 × 10^9^/L CRP 213 mg/L	Osteomyelitis after 2 aseptic revision procedures followed by below knee amputation	3
11	Ankle	79 M	37	Congestive cardiac failure, gout, previous alcohol excess, left THR, right hip hemiarthroplasty, peripheral neuropathy, BPH	WCC 25.2 × 10^9^/L CRP 181 mg/L	Sepsis secondary to osteomyelitis after 1^st^ revision procedure requiring a below knee amputation	2
18	Quadriceps tendon	65 M	39	HTN	WCC 6.9 × 10^9^/L CRP 208 mg/L	Infection noted after revision. Required 3 washouts after deep infection. Further revision required due to gapping in tendon. Discharged with satisfactory function	4
22	Ankle	55F	28	Alcohol excess, liver disease, encephalopathy, overdose, peripheral neuropathy, chronic pain	WCC 11.6 × 10^9^/L CRP 218 mg/L	Managed with Ilizarov frame following failed 1^st^ revision surgery. Below knee amputation due to complex regional pain syndrome	3
23	Ankle	46F	40	Depression, personality disorder, fatty liver, alopecia	N/A (low grade infection diagnosed from tissue culture)	2 revision operations. 1^st^ for syndesmosis screw removal due to lysis and 2^nd^ for medial malleolus non-union revision fixation. Discharged	2
33	Ankle	48F	42	Asthma, recurrent chest infections, hiatus hernia, previous anxiety and depression	WCC 18.5 × 10^9^/L CRP 203 mg/L	Infection post initial revision managed initially with a bridging external fixator. This was followed by fusion procedure using Iliazrov frame. Deceased with cause of death unrelated to orthopaedic diagnosis	3
34	Ankle	55 M	23	Alcoholic liver disease, hepatic encephalopathy, oesophageal varices, duodenal ulcer, ascites, ibs, chronic bilateral foot pain, depression & anxiety	WCC 14.7 × 10^9^/L	Failed ankle fixation revised. Infection noted after revision and subsequently managed with a hindfoot nail. Deceased with cause of death unrelated to orthopaedic diagnosis	2
40	Subtrochanteric hip	54 M	30	Alcohol excess, chronic venous insufficiency, HTN	WCC 7.6 × 10^9^/L CRP 14 mg/L	Revised to blade plate for aseptic non-union. Infection noted thereafter and required one washout. Discharged. Deceased with cause of death unrelated to orthopaedic diagnosis	2
44	Subtrochanteric hip	90F	16	Falls, dementia, anxiety and depression, rheumatoid arthritis, vitamin B12 deficiency, barrett's oesophagus	WCC 19.3 × 10^9^/L CRP 252 mg/L	Femoral IM nail followed by failure of implant after further trauma. Revised to hemiarthroplasty which acquired an infection. Proceeded to excision arthroplasty	2

*HTN* Hypertension, *IBS* Irritable bowel syndrome, *THR* Total hip replacement, *BPH* Benign prostatic hypertrophy, *IM* Intramedullary, *WCC* White cell count, *CRP* C-reactive protein

### Statistical analysis

Statistical analysis was performed using Statistical Package for Social Sciences version 17.0 (SPSS Inc., Chicago, IL, USA). A Student’s t-test was used to compare parametric data whilst dichotomous variables were assessed using a Chi square test. Fishers exact test was used for categorical data with five or less in one of the cells. Multivariable logistic regression analysis was used to assess the independent association of factors associated with infection after aseptic revision when adjusting for confounding variables. Sample size calculation for the logistic model was limited due to the small number of patients in the infection group (*n* = 15) and therefore forward and backward conditional modelling was performed to reject variables that were not significant [13]. A *p*-value ≤ 0.05 was defined as significant.

## Results

Eighty-six patients were identified as meeting the inclusion criteria, with a mean age of 53 (range14–95) years and 48 (55.8%) were female. The mean total follow up was 3.1 (range 2.1–4.3) years. Fifteen (17%) patients acquired either a superficial (*n* = 6, 7%) or deep (*n* = 9, 10%) infection following aseptic revision surgery. Forty-three (43/86, 50%) patients had a simple removal of metalwork, of which 3 (7%) acquired a superficial infection post-operatively only. All six patients with superficial infections made an uneventful recovery following a course of oral (*n* = 4) or intravenous (*n* = 2) antibiotics and no further surgery was required at the time of last review. There were nine deep infections that required re-revision surgery (Table 1). The morbidity associated with a deep infection following re-revision was significant, with only three patients being discharged, whilst another three patients underwent a below knee amputation (Table 1).

### Factors associated with SSI

There were no differences in sex (*p* = 0.719, chi-square), BMI (*p* = 0.262, independent *t*-test), Scottish Index of Multiple Deprivation (*p* = 0.359, chi square), smoking status (*p* = 0.906, Fishers exact test), intra-articular involvement (*p* = 0.853, chi square between) or limb involvement (*p* = 0.129, Fisher’s exact test) between those with and without an infection following revision fixation (Table 2). There was a trend towards a significance association for infection with older age (*p* = 0.084, independent t-test), dementia (*p* = 0.079, Fisher’s exact test) and liver disease (*p* = 0.079, Fisher’s exact test) (Table 2). A high alcohol intake (*p* = 0.046, chi square), and comorbidities of chronic obstructive pulmonary disease (COPD) (*p* = 0.019, Fisher’s exact test) and CVA/TIA (*p* = 0.029, Fisher’s exact test) were associated with a significantly increased chance of infection following revision surgery (Table 2). Increased time from initial injury to primary fixation showed a significant increase risk for acquiring an infection post revision trauma surgery (*p* = 0.007), but the time between primary fixation and revision surgery did not show a difference (*p* = 0.381) (Table 2). When adjust for confounding factors on regression analysis a high alcohol intake (OR = 1.61, 95% CI 1.01–6.36, *p* = 0.046) and a diagnosis of COPD (OR = 11.1, 95% CI 1.00–133.3, *p* = 0.05) were independently associated with the risk of acquiring an infection (Table 3).

**Table 2:** Patient demographics, lifestyle data and co-morbidities of patients undergoing a revision trauma operation

Demographic	Descriptive	Group		Odds ratio/difference (95% CI)	*p*-value
		No infection (*n* = 71)	Infection (*n* = 15)		
Sex (M/F) (*n*, % of group)	Male	32	6	1.23 (0.40–3.835)	0.719*
	Female	39	9		
Mean age (years: mean, SD)		52.1 (22.5)	60.9 (16.0)	Diff 8.8 (− 1.3–18.9)	0.084**
SIMD	1 (most deprived)	8	2	N/A	0.359*
	2	18	3		
	3	9	5		
	4	17	3		
	5 (least deprived)	19	3		
BMI (Kg/m^2^: mean, SD)		27.6 (5.4)	29.6 (8.0)	Diff 2.0 (− 1.6 to 5.7)	0.262**
Smoker	Yes	20	4	0.93 (0.26–3.26)	0.906***
	No	51	11		
Alcohol intake		59	9	3.28 (1.00–10.94)	0.046*
		12	6		
Comorbidity	IHD/HF	2	2	5.31 (0.69–41.13)	0.079***
	CVA/TIA	0	1	–	0.029***
	COPD	1	3	17.50 (1.68–182.50)	0.002***
	Diabetes	5	1	0.94 (0.10–8.71)	0.959***
	Connective Tissue	4	2	2.58 (0.43–15.56)	0.288***
	Dementia	2	2	5.31 (0.69–41.13)	0.079***
	Liver disease	2	2	5.31 (0.69–41.13)	0.079***
	Kidney disease	1	0	–	0.644***
	Gastric ulcer	2	1	2.46 (0.21–29.08)	0.460***
	Tumour	3	0	–	0.418***
	Immune suppression	4	2	2.58 (0.43–15.56)	0.288***
Intra-articular	No	35	7	1.11 (0.36–3.39)	0.853*
	Yes	36	8		
Time (median)	Injury to 1st op	1 (1–6)	1 (1–2)		0.015****
	1st–2nd op	60 (22–60)	33 (18–135)		0.189****
Limb	Upper	29	3	2.76 (0.72–10.66)	0.129***
	Lower	42	12		

*Chi square test unless **Independent *t*-test ***Fisher’s exact test ****Mann–Whitney *u*-test

**Table 3:** Logistic regression analysis of preoperative variables associated with infection in patients undergoing revision fracture fixation. Nagelkerke R2 0.395

Preoperative variable		Odds ratio	95% CI	*p*-value
Alcohol	No	Reference		
	Yes	1.61	1.01–6.36	0.046
COPD	Yes	Reference		
	No	11.1	1.00–133.3	0.050

*Odds ratio for each increasing year of age

## Discussion

The current study has shown that aseptic revision surgery for orthopaedic trauma was associated with a high risk (17%) of infection, which was life changing for patients developing a deep infection (10%), with significant persistent morbidity. Approximately one in six patients undergoing aseptic revision fixation surgery acquired a surgical site infection. All deep infections were found to be in patients with lower limb injuries. This is almost six times higher than an estimated risk for infection for primary trauma operations which is reported to be between two and four percent [14, 15]. Independent risk factors associated with surgical site infection following aseptic revision fixation were a high alcohol intake and a comorbidity of COPD.

The rate of infection following primary trauma orthopaedic surgery ranges between 1 and 3% [16]. However, the reported rate of surgical site infection (SSI) does vary throughout the literature and the incidence has been quoted to be as high as 50% for some ‘at risk’ anatomical areas, such as the tibial plateau and pilon type fractures [16, 17]. A systematic review of 10 studies specific to infections after primary ankle fracture fixation had an incidence of 7%, which is high [18]. The same systematic review which included over 8000 ankle fractures found that high BMI, American Society of Anesthesiologists grade three or more, diabetes, alcohol, subluxation/dislocation, high energy mechanism and heart failure were all risk factors for infection following fixation. [18] Alcohol excess in particular has been found to be a risk factor for infection in primary fixation procedures of the ankle with the hypothesis that alcohol excess reduces the host immune system capacity pre-disposing patients to complications, such as surgical site infections [19, 20]. This was also affirmed in the current study, with high alcohol intake being independently associated with an increased risk of surgical site infection following aseptic revision surgery. High BMI is also noted to contribute to primary surgical site infection in orthopaedic patients undergoing acetabular fracture fixation and elective joint arthroplasty surgery [21, 22]. This is attributed to the systemic inflammatory state seen in patients with a high BMI, lipid dysregulation or diabetes [23]. However, the current study did not demonstrate BMI to be a risk factor for infection following revision fixation surgery, but this may be due to a type two error from the small cohort and the two point BMI difference between the infected and non-infected patients being non-significant. A novel aspect of the current study was identification of COPD as an independent risk factor for infection, but the reason for this is not clear. COPD is a recognised risk factor for deep infection following an open tibial shaft fracture [24] and serious infection following hip and knee arthroplasty [25]. It is hypothesised that the reason for this association may be related to perioperative hypoxia and/or steroid use.

We report a high rate of deep surgical site infection in revision trauma surgery which was shown to result in serious potential outcomes including amputation. Three patients in the current study underwent a lower limb amputation as a result of deep infection following their aseptic revision procedure. The authors feel this is of great value in setting surgeons’ and patients’ expectations peri-operatively and to our knowledge this has not been published before. Another interesting finding highlighted in the case series of nine patients is the number of operations undertaken as salvage procedures for failed fixation. Twenty-three operations were undertaken for just nine patients after failed primary fixation. In fact, after the first revision, a total of 14 procedures were undertaken as salvage procedures prior to final outcome for these nine patients. This number is a conservative figure as it can be noted that three patients died during the management of their failed fixation procedure. The cost burden of these revision procedures, the hospital overnight stay and outpatient clinics on the National Health Service would be extremely high. However, the current study has not assessed the success of revision procedures in detail and it is therefore uncertain and whether revision surgery is cost effective.

In conclusion, aseptic revision trauma surgery had a high incidence of subsequent infection. Complications were associated with a diagnosis of COPD. Deep surgical site infection occurred in 11% of patients, therefore, patients undergoing revision fixation should be informed of this as part of the consent process. Revision of trauma operations, particularly around the ankle, carries a significant risk and the benefits of embarking on such surgery should be carefully weighted against these risks.

### Limitations

There are a number of limitations of the current study that need to be acknowledged. Firstly, this is a retrospective study design and therefore the accuracy of data collected was reliant on information collected by clinicians at the time of injury, operation and follow up. Patient reported outcome measures (PROMS) were not obtained and therefore we cannot make any objective assessments regarding functional limitations or quality of life following SSI after revision surgery. Another limitation is that our study has a small sample size as operations for aseptic revision surgery are relatively uncommon. The current study collected data retrospectively over a 3-year period and found an incidence of only 30 cases per year. The study centre performed 10,597 trauma operations during the 3-year study period; therefore the rate of aseptic revision fixation was relatively low (86 cases). It would be difficult to carry out a prospective study on this topic in view of the low incidence.

### Future directions

Further research regarding infections post trauma revision surgery is required. Ideally this would be designed as a prospective series with a protocol in place regarding diagnosis, tissue sampling and follow up.
